# Topiramate Add-on Lithium Carbonate for Treatment of Acute Mania

**Published:** 2013

**Authors:** Zahra Mirsepassi, Robabeh Mazinani, Farbod Fadai, Neda Alibeigi, Ali Nazeri Astaneh

**Affiliations:** 1 Resident, Department of Psychiatry, Razi Psychiatric Hospital, University of Social Welfare and Rehabilitation Sciences, Tehran, Iran.; 2 Psychiatrist, Associate Professor, Department of Psychiatry, Razi Psychiatric Hospital, University of Social Welfare and Rehabilitation Sciences, Tehran, Iran.; 3 Clinical Psychologist, Assistant Professor, Department of Psychiatry, Razi Psychiatric Hospital, University of Social Welfare and Rehabilitation Sciences, Tehran, Iran.; 4 Psychiatrist, Assistant Professor, Department of Psychiatry, Razi Psychiatric Hospital, University of Social Welfare and Rehabilitation Sciences, Tehran, Iran.

**Keywords:** Lithium, Topiramate, Mania, Treatment

## Abstract

**Objective:** The purpose of this study was to evaluate the effect of adjunctive topiramate in treating acute mania.

**Methods:** In this study which was a double-blinded randomized clinical trial, 46 bipolar patients in manic episode, were treated with lithium carbonate and topiramate versus lithium carbonate and placebo and treatment responses were assessed by Young Mania Rating Scale (YMRS) weekly.

**Results: **In both intervention and control groups, YMRS score had significant decline after 8 weeks(p < 0.001), but there was no statistically significant difference between the two groups (p = 0.419). The highest score decline was after two weeks. YMRS score at baseline did not have statistically significant difference between the intervention and control groups (p = 0.709).

**Conclusions:** This study failed to show antimanic efficacy of adjunctive topiramate in the treatment of those with acute manic.

**Declaration of interest:** None.

**Clinical Trial Registration:** URL: http://www.irct.ir.IRCT201110317960N1.

## Introduction

Bipolar disorder is a common psychiatric disorder and it may cause disability in patients. Although different medications are effective in treatment of acute mania, some patients are resistant to conventional treatments (1-3). Lithium, sodium valproate and carbamazepine are Food and Drug Administration (FDA) approved for the treatment of acute mania, and concurrent administration of atypical antipsychotics and benzodiazepines would be more effective (2). 

In preliminary studies, response rate to lithium was 70% but recent studies showed only 50% (2). All antipsychotics (typical and atypical) are effective in acute mania (2). Amongst anticonvulsants, some are effective and some are not. Topiramate is an anticonvulsant, its mechanism of action is unknown but it is known to enhance the activity of gamma-aminobutyric acid (GABA), and block the effect of glutamate at N-methyl-D-aspartate (NMDA) receptors and inhibits some isoenzymes of carbonic anhydrase (CA-II and CA-IV). In addition, there is evidence that topiramate may also possess a state-dependent sodium channel blocking action in neurons (4). 

There are some controversies about the effectiveness of this medication in acute mania (5-15). Limitation of these studies may play a role on the results. Topiramate is safe and well tolerated medication and its adverse effects are often mild to moderate, mostly in the first month of treatment and will be resolved after discontinuation. Decreased appetite and weight loss are common side effects of topiramate and these side effects profile would be useful in manic patients, who are at high risk of weight gain because of the adverse effects of mood stabilizers and antipsychotics.

To our knowledge, only two studies have been conducted in Iran about topiramate in treatment of bipolar disorder, which is concerned with children and adolescents and not adults (16, 17). The present study aimed to evaluate the antimanic effect of topiramate as an adjunctive treatment to lithium in adults.

## Materials and Methods

Patients, aged 18 to 65, suffering from bipolar I disorder most recent manic episode hospitalized in Razi Psychiatric Hospital (Tehran, Iran), were selected via convenient sampling method and then were randomly divided into two groups i.e. control and intervention. 

The exclusive criteria were pregnant and lactating women, patients with disabling medical or neurological disorders, substance related disorders, history of kidney disease or renal stone and history of hypersensitivity to topiramate. 

The study was approved by the Ethics Committee of Razi University of Social Welfare and Rehabilitation Sciences. The possible risks and benefits of treatment with topiramate were explained to the patients and relatives and a written informed consent was obtained from them. 

Using Cohen Statistical Power Analysis, 23 patients allocated to each group in a double-blinded set. In the intervention group, patients received 900 mg of lithium carbonate and 2 mg of risperidone per day (increased to 8 mg per day if needed) or equivalent dose of other antipsychotics plus topiramate starting with 50 mg per day and increasing the dosage 50 mg each 3 days to reach the maximum dosage of 200 mg per day (4-6). 

In the control group, patients received 900 mg of lithium carbonate and 2 mg of risperidone per day (increased to 8mg per day if needed) or equivalent dosage of other antipsychotics plus placebo. Patients were interviewed weekly by a penultimate psychiatric trained resident for this purpose in a blinded set and the response to treatment was assessed at the baseline and weekly thereafter, using Young Mania Rating Scale (YMRS). 

YMRS consists of 11 items based on core symptoms of mania. Four items (irritability, speech, thought content, and impulsivity) scored 0, 2, 4, 6, 8 and others (elated mood, psychomotor agitation, appearance, insight, form of thought, and sleep) scored 0, 1, 2, 3, and 4. Barekatain et al. reported a good validity and reliability for the Persian version of this scale (18). 

Medication compliance, appetite, weight, suicidal thoughts, side effects of topiramate and symptoms and signs of lithium toxicity were assessed in every interview. In the first visit, the height and weight measured and accordingly body mass index (BMI) was recorded. Duration of the study took 6 weeks after reaching the optimal dosage of topiramate which was 200 mg per day (8 weeks after onset of the study).

## Results

Demographic characteristics of the participants are shown in table1. YMRS score at baseline did not show statistically significant difference between the intervention and control groups (p = 0.709). In both groups, YMRS score had significant decline after 8 weeks (p < 0.001), but there was no statistically significant difference between the two groups (p = 0.419).The highest score decline within the groups, was after two weeks.

Amongst 23 patients in the intervention group, 16 patients received risperidone, 4 patients received olanzapine, 2 patients received quetiapine, and only one patient received aripiprazole. No patient in the intervention group needed to receive clozapine. The mean dosage of antipsychotics in this group was 6 mg for risperidone, 15 mg for olanzapine, 175 mg for quetiapine and 30 mg for aripiprazole. 

Amongst 23 patients in the control group, 16 received risperidone, 6 received olanzapine and one received quetiapine. No patient received clozapine or aripiprazole in this group. 

**Table 1:** Demographic characteristics of patients in both groups (intervention and control)

**Variable**	**Control (n = 23)**	**Intervention (n = 23)**	**P value**
**Sex**			
**Female**	11 (47.8)	11 (47.8)	0.616
**Male**	12 (52.2)	12 (52.2)	
**Age**	40.4 +-9.4	35.6+-6.8	0.720
**Marital status**			
**Single**	05 (21.7)	11 (47.8)	
**Married**	14 (60.9)	10 (43.5)	0.167
**Divorced**	04 (17.4)	2 (8.7)	
**Education**			
**Under high school graduate**	2 (8.7)	10 (43.5)	0.376
**High school Diploma**	11 (47.8)	10 (43.5	
**University degree**	09 (39.1)	003 (13.0)	
**Occupation**			
**Unemployed**	07 (30.4)	11 (47.8)	0.495
**Homemaker**	09 (39.1)	09 (39.1)	
**Self employed**	05 (21.7)	03 (13.0)	
**Governmental employee**	1 (4.3)		
**Retired**	1 (4.3)		

The mean dosage of antipsychotics in this group was 5.2 mg for risperidone, 15 mg for olanzapine and 175 mg for quetiapine. Furthermore, the dosage of benzodiazepines did not show statistically significant difference between the two groups.

In intervention and control groups, appetite did not show statistically significant difference after 8 weeks (p = 0.429 and 0.713, respectively). At the baseline, there was no statistically significant difference between two groups as far as weight was concerned (p = 0.948). There was also no statistically significant difference in weight, after 8 weeks, within the intervention and control (p = 0.123 and 0.944, respectively) and between two groups (p = 0.254). 

Five patients were discharged at the first week of admission due to personal request. All these patients were in control group. One patient from control group and one patient from intervention group did not follow the treatment after 3 and 4 weeks, respectively. One patient from placebo group discontinued the study because of creatinine rise and then received sodium valproate and after this change, the creatinine level turned to the normal level. One patient from placebo group discontinued the study after 3 weeks because of neuroleptic malignant syndrome and received a relevant treatment. Three patients -one from intervention and two from control group- discontinued the study as a result of showing no response to treatment after 6 weeks and received electro convusive therapy (ECT). 

Finally 34 patients completed the study (21 patients from intervention and 13 from control). Data of all patients, including those who could not completed our study, were used in our statistical analysis.

## Discussion

Bipolar disorder is a common psychiatric disorder and can cause disability in patients. Some bipolar patients in manic episode are resistant to conventional treatments. In recent studies, response rate to mood stabilizers monotherapy and antipsychotics alone was about 50%. It can be increased to 60-80% when one mood stabilizer and one antipsychotic are administered together (2). These findings demonstrated that adjunctive therapy in treating acute mania could be helpful (2). So, we chose topiramate as a new generation anticonvulsant, with a favorable pharmacokinetic and side effects profile, as an adjunctive treatment to lithium carbonate for our study (1, 2, 4, 6, 13). 

Our study, which was carried out to evaluate the efficacy of lithium carbonate and topiramate versus lithium carbonate and placebo in bipolar patients in manic episode, failed to show antimanic efficacy of adjunctive topiramate in manic patients. Our results were similar to some of previous studies (13, 14, 15).

In an open label trial, Bozikas et al. showed a modest efficacy of topiramate, especially as monotherapy, in the treatment of acute mania (13). In a randomized clinical trial, Roy Chengappa et al. showed that there was no difference in the reduction of YMRS score in topiramate and placebo groups and both groups showed 40% declines (14). In a review, Goodnick noted that topiramate failed in large-scale investigations in bipolar disorder (15). In our study, both groups showed decline in YMRS scores from the first week and the most therapeutic effects appeared between weeks 2 and 8. This finding was in accordance with Vieta (11). 

Our findings did not approve the results of some preliminary studies which showed that topiramate may be a useful medication in the treatment of mania. Most of these preliminary studies had important limitations such as open label design (5-7, 9-12, 17), small sample size (9, 11, 13, 17), retrospective manner of study (5, 9, 10), nonrandomized (5, 6, 11) and uncontrolled design (9, 10, 11, 13, 17). 

Although our sample size was small and this was an important limitation in our study, it had also some advantages; it was a double-blinded randomized clinical trial, and to our knowledge, this is the first study in Iran, to evaluate the antimanic efficacy of topiramate in adults. The previous studies in Iran had been conducted in children and adolescents (16, 17).

Suicidal thought is one of the important side effects of anticonvulsants medications, such as topiramate (4). In our study, no patient had suicidal ideas as the side effects of topiramate treatment. Topiramate was well tolerated in all patients and no one discontinued the study due to the side effects of medication. 

The results of this study should be interpreted with caution because of a number of limitations. First, the sample size was small which might cause statistically non-significant (NS) difference. Second, duration of follow-up was only 8 weeks. Third, in this study, benzodiazepines and different type of antipsychotics with different doses were administered if needed and finally high rate of dropouts was another point that should be taken into consideration. 

## Conclusion

This study failed to show antimanic efficacy of adjunctive topiramate in the treatment of acute manic patients.

Studies with larger sample size, longer period of follow-up, no concomitant use of benzodiazepines and antipsychotics or use of a certain antipsychotic with fixed dosage in all participants and rather higher doses of topiramate are recommended.

## Authors' contributions

ZM and RM conceived and designed the evaluation and helped to draft the manuscript. FF and AN participated in designing the evaluation and re-evaluated the clinical data and revised the manuscript. NA performed the statistical analysis and revised the manuscript. ZM collected the clinical data, interpreted them and revised the manuscript. ZM and RM re-analyzed the clinical and statistical data and revised the manuscript. All authors read and approved the final manuscript.
